# Genome-Wide DNA Methylation Signatures of Sea Cucumber *Apostichopus japonicus* during Environmental Induced Aestivation

**DOI:** 10.3390/genes11091020

**Published:** 2020-08-31

**Authors:** Yujia Yang, Yingqiu Zheng, Lina Sun, Muyan Chen

**Affiliations:** 1 Laboratory for Evolution and Development, Institute of Evolution and Marine Biodiversity, Ocean University of China, Qingdao 266003, China; yuzhongjiaren@126.com; 2 The Key Laboratory of Mariculture, Ministry of Education, Ocean University of China, Qingdao 266071, China; yingqiuz@163.com; 3 CAS Key Laboratory of Marine Ecology and Environmental Sciences, Institute of Oceanology, Chinese Academy of Sciences (CAS), Qingdao 266003, China

**Keywords:** marine invertebrates, epigenetics, environmental induced hypometabolism, phenotypic plasticity, methylation-related enzymes

## Abstract

Organisms respond to severe environmental changes by entering into hypometabolic states, minimizing their metabolic rates, suspending development and reproduction, and surviving critical ecological changes. They come back to an active lifestyle once the environmental conditions are conducive. Marine invertebrates live in the aquatic environment and adapt to environmental changes in their whole life. Sea cucumbers and sponges are only two recently known types of marine organisms that aestivate in response to temperature change. Sea cucumber has become an excellent model organism for studies of environmentally-induced aestivation by marine invertebrates. DNA methylation, the most widely considered epigenetic marks, has been reported to contribute to phenotypic plasticity in response to environmental stress in aquatic organisms. Most of methylation-related enzymes, including DNA methyltransferases, Methyl-CpG binding domain proteins, and DNA demethylases, were up-regulated during aestivation. We conducted high-resolution whole-genome bisulfite sequencing of the intestine from sea cucumber at non-aestivation and deep-aestivation stages. Further DNA methylation profile analysis was also conducted across the distinct genomic features and entire transcriptional units. A different elevation in methylation level at internal exons was observed with clear demarcation of intron/exon boundaries during transcriptional unit scanning. The lowest methylation level occurs in the first exons, followed by the last exons and the internal exons. A significant increase in non-CpG methylation (CHG and CHH) was observed within the intron and mRNA regions in aestivation groups. A total of 1393 genes were annotated within hypermethylated DMRs (differentially methylated regions), and 749 genes were annotated within hypomethylated DMRs. Differentially methylated genes were enriched in the mRNA surveillance pathway, metabolic pathway, and RNA transport. Then, 24 hypermethylated genes and 15 hypomethylated genes were Retrovirus-related Pol polyprotein from transposon (RPPT) genes. This study provides further understanding of epigenetic control on environmental induced hypometabolism in aquatic organisms.

## 1. Introduction

Changing environmental conditions is a challenge for virtually all organisms. It may be caused by predictable rhythms (e.g., daily, tidal, seasonal) or episodically (e.g., a sudden environmental change in temperature, water, oxygen, nutrients, etc). An organism can adapt to a unique environment and change its behavior, morphology, and physiology [1]. Many species enter into environmental induced hypometabolism (e.g., hibernation, aestivation, or diapause) in response to predictable environmental signals [2,3,4]. This environmental induced hypometabolism shows reversible phenotypic plasticity, which happens seasonally but reversibly [5,6,7].

Epigenetic modifications are heritable alterations without changes in DNA sequences, which alter DNA accessibility and chromatin structure and regulate gene expression patterns in many biological processes (e.g., development, differentiation, disease, aging, and immunity) [8,9]. In recent years, these stable epigenetic marks have also been proposed to be involved in phenotypic plasticity and contribute to an organism’s ability to respond to external environmental triggers [10,11]. When organisms enter into hypometabolic states, epigenetic mechanisms might play an important role in global transcriptional suppression [12,13]. Recent studies have confirmed that epigenetic modifications contributed to transcriptional control over cycles of torpor-arousal during hibernation in thirteen-lined ground squirrels, *Ictidomys tridecemlineatus* [14]. Epigenetic mechanisms could contribute to gene-environment interaction and potentially inherited impacts on the phenotype and short- and long-term adaptation of marine organisms [15].

DNA methylation, the most widely studied epigenetic marks, has been reported to contribute to phenotypic plasticity in response to environmental stress in aquatic organisms, especially marine invertebrates [16,17]. Genomic DNA methylation can globally regulate gene expression, thereby mediating energy efficiency during a hypometabolic state [13]. The molecular basis during hypometabolic conditions in marine invertebrates is still understudied and is only beginning to be explored. Limited research in *Crassostrea gigas* suggested that DNA methylation has regulatory functions in stress and environmental responses, particularly in gene families with inducible expression [17]. In corals, strong negative responses and DNA methylation variation were observed in response to ocean acidification [18]. The phenotypic plasticity generated by DNA methylation might be potentially heritable for genetic adaptation and evolution [18].

Aestivation, a form of aerobic dormancy, is a widespread response to adverse environmental conditions among vertebrate and invertebrate species [3]. Aestivation can be caused by a circannual rhythm (hot arid summer conditions) or a sudden environmental change such as temperature variation or absence of food and water [19]. Sea cucumber, *Apostichopus japonicus*, lives in shallow temperate waters along the coasts and is susceptible to environmental changes such as temperature and salinity fluctuations [20]. In summer, when the temperature of seawater rises to a certain level, most individuals enter into aestivation with minimal activity and depressed metabolism, lasting up to 100 days [21]. The digestive tract showed phenotypic plasticity during aestivation, as the bodyweight and digestive enzyme activities recovered [22]. The life stages could be divided into four stages by the physiological states: active stage, prophase of inactivity, peak inactivity, and reversion phase [22].

In recent years, several studies have focused on DNA methylation regulation on aestivation in sea cucumbers. Zhao et al. first reported that DNA methylation occurred in various sea cucumber tissues during aestivation using F-MSAP (fluorescence-labeled methylation-sensitive amplified polymorphism) analysis, and the primary modification tissues are intestine and the respiratory tree of sea cucumbers. Follow-up research was focused on the relationship between DNA methylation and cell cycle regulator, cyclin B, during aestivation [23]. In a recent study, RNA-seq and MethylRAD analysis revealed the transcriptional network and regulation mechanism of intestine hypometabolism during aestivation [24].

Metabolic suppression during aestivation requires coordinated regulation of multiple cellular processes potentially through epigenetic modification and gene transcription. Determining the functional significance of global DNA methylation in sea cucumbers will prove valuable for understanding seasonal temperature variation-induced hypometabolism in aquatic organisms. In the present study, we reported a genome-wide examination of DNA methylation in the intestine of sea cucumber *A. japonicus* during thermal stress-induced hypometabolism. We conducted high-resolution whole-genome bisulfite sequencing of intestine from sea cucumber during non-aestivation (control group) and deep-aestivation stages (aestivation group) to investigate the global DNA methylation profiling. This study provided a further understanding of DNA methylation modification on seasonal temperature-induced aestivation in aquatic organisms.

## 2. Materials and Methods

### 2.1. Sample Collection and Preparation

Healthy adult sea cucumbers *A. japonicus*, wet body weight 100 ± 10 g, were collected from the coast of Jiao Zhou Bay of the Yellow Sea, Qingdao, Shandong Province, China. The control groups (Sample C1–C3) were sampled in May when sea surface temperature was approximately 15 °C. These individuals have gone through the aestivation period and fully recovered to their active status for several months. Aestivation groups (Sample A1–A3) were sampled in mid-August when sea surface temperature was approximately 26 °C. The individuals from the aestivation group were collected after 15 days of continuous aestivation as indicated by cessation of feeding, locomotion, and intestine degeneration into a very tiny string (approximately 2 mm) [25]. Nine individuals (three biological replicates × three sea cucumbers per biological replicate) from control and aestivation groups were used. The intestine tissues were dissected and washed, flash-frozen in liquid nitrogen, and kept at −80 °C freezer for genomic DNA and RNA extraction.

### 2.2. Total RNA Extraction and Quantitative RT-PCR

The intestine tissues were sampled from control and aestivation groups (three replicates, three individuals/replicate). The samples used for qRT-PCR were sampled at the same time when we collected samples for WGBS. Total RNA was isolated using the RNeasy Mini Kit (Cat No. 74104, QIAGEN, Hilden, Germany) following the manufacturer’s instructions. RNA concentration and quality were determined using NanoDrop1000 (Thermo, Rockford, MA, USA). First-strand cDNA was synthesized using PrimeScript™RT Kit with gDNA Eraser (Code No. RR047A, Takara, Kusatsu, Shiga, Japan) as qRT-PCR templates. Thermal cycling was as follows: 37 °C for 15 min, 85 °C for 5 s, stop at 4 °C. The primers for qRT-PCR were designed for DNA methylation-related genes using Primer 3 (http://frodo.wi.mit.edu/). The sequences of primers were listed in Table 1.

Quantitative RT-PCR was performed on an SYBR Green real-time PCR assay (SYBR PrimeScriptTM RT-PCR Kit II, TaKaRA) with an Eppendorf Mastercycler ep realplex (Eppendorf, Hamburg, Germany). Then, 40 S ribosomal protein S18, ß-tubulin, and NADH dehydrogenase were used as internal controls, as described previously [26]. In our study of reference gene stability [27], the expression of these reference genes was stable during aestivation in the intestine tissue of *A. japonicus*. Thermal cycling was as follows: 95 °C for 5 s, 40 cycles at 95 °C for 10 s, 60 °C for 20 s and 72 °C for 30 s. The 2^−ΔΔCT^ method was used to analyze the expression levels, and the target genes’ Cts were normalized using the geometric mean of these three internal control genes. The relative expression levels were shown as mean ± s.d., and the statistical significance was set at *p* < 0.05.

### 2.3. Genomic DNA Extraction and Whole-Genome Bisulfite Sequencing

Genomic DNA was extracted from intestine tissues (~25 mg) of *A. japonicus* using DNeasy Blood and Tissue Kit (Cat No. 69504, QIAGEN) following the manufacturer’s protocol. Genomic DNA of intestine tissues from control and aestivation groups was sent to BGI (BGI Tech Solutions Co., Ltd., Shenzhen, China) for whole-genome bisulfite sequencing. Genomic DNA was firstly fragmented by sonication using a Bioruptor to a mean size of approximately 250 bp, followed by the blunt-ending, dA addition to 3’-end, and adaptor ligation. Ligated DNA was bisulfite converted using the EZ DNA Methylation-Gold kit (ZYMO, Irvine, CA, USA). Further, fragments with a length of 100–300 bp were selected and purified by the QIAquick Gel Extraction kit (QIAGEN). The products were then amplified by PCR. At last, we performed 100 bp paired-end sequencing on HiSeq 2000 Sequencing System. Sequence data have been deposited at the NCBI BioProject database under accession PRJNA643989.

### 2.4. Identification of Differentially Methylated Regions

The raw reads were filtered to remove the adaptors, contamination, and low-quality data (unknown bases more than 10%; the ratio of bases whose quality was less than 20). The clean bisulfite reads were mapped to the sea cucumber reference genome [28] using BSMAP [29], and the duplicated reads were removed. The BSMAP script was BSMAP -a filename_1.clean.fq.gz -b filename_2.clean.fq.gz -o filename.sam -d ref.fa -u -v 8 -z 64 -*p* 4 -n 0 -w 20 -s 16 -f 10 -L 125. The sam files were converted to bam files using scripts (samtools view -S -b -o filename.bam filename.sam; samtools sort -m 2000000000 filename.bam filename.sort.bam; samtools index filename.sort.bam). The mapping rate and bisulfite conversion rate of each sample were calculated. The CpG methylation clustering and Principal Component Analysis were generated by MethylKit [30]. The scripts we used were clusterSamples(meth, dist = "correlation", method = "ward", plot = TRUE) and PCASamples(meth). The methylation level was determined by dividing the number of reads covering each mC by the total reads [31], which equal the mC/C ratio at each reference cytosine [32]. The formula we used was $Rm_{averge}=\frac{Nm_{all}}{Nm_{all}+Nnm_{all}}$. Nm represents the reads number of mC, while Nnm represents the reads number of non-methylation reads. Differentially methylated regions (DMRs) were identified by windows that contained at least 5 CpG (CHG or CHH) sites with a 2-fold change in methylation level and Fisher’s test *p* ≤ 0.05. The heat maps were built to present the distribution of methylation levels in distinct genomic features [33]. Canonical DNA methylation profiles of the entire transcriptional units were divided into different functional elements [33] to study the methylation level changes.

### 2.5. Gene Ontology and Pathway Enrichment of DMRs

GO enrichment analysis provided GO terms that were significantly enriched in the list of DMR-related genes, and filtered DMR-related genes that correspond to specific molecular function, cellular component, and biological processes. The DMR-related genes were mapped to the GO database (http://www.geneontology.org/). The significant enriched GO terms were identified using the hypergeometric test, and the calculated *p*-value goes through Bonferroni Correction, taking corrected *p* ≤ 0.05 as a threshold. KEGG pathway enrichment analysis further identified significantly enriched metabolic pathways or signal transduction pathways and helped understand the biological functions of the DMR-related genes [34]. The calculation is the same as that in the GO analysis.

## 3. Results

### 3.1. Expression of DNA Methylation Related Enzymes during Aestivation

**Figure 1:**
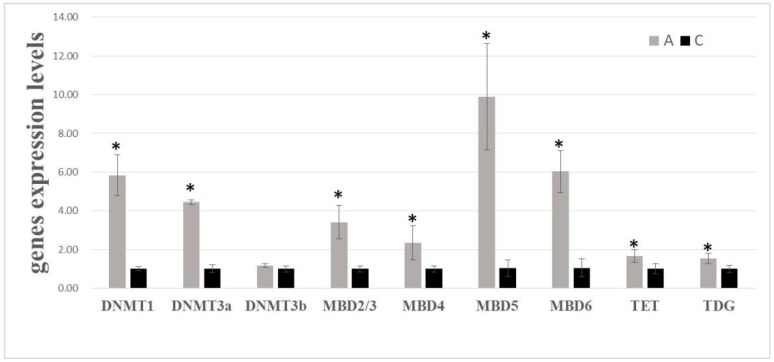
Gene expression levels of DNA methyltransferases, Methyl-CpG binding domain proteins, and DNA demethylase in sea cucumber intestine during aestivation. Significant levels are indicated by * (*p* < 0.05).

Quantitative RT-PCR was used to specifically investigate the gene expression levels of DNA methyltransferases (DNMT1, DNMT3a, DNMT3b), Methyl-CpG binding domain proteins (MBD2/3, MBD4, MBD5, MBD6), and DNA demethylases (TET, TDG). Despite DNMT3b, all target genes were up-regulated during aestivation (Figure 1). More specifically, DNMT1 and DNMT3a were highly expressed in aestivation groups with a fold change of around six and four times. DNMT3a showed no differences between control groups and aestivation groups. All MBD proteins showed higher expression levels in aestivation groups, with a fold change of around three times (MBD2/3), twice (MBD4), ten times (MBD5), and six times (MBD6). TET and TDG were both expressed with a 1.5 fold change in aestivation groups.

### 3.2. DNA Methylation of Sea Cucumber during Aestivation

We conducted whole-genome bisulfite sequencing of sea cucumber intestine from aestivation groups and control groups. After quality control of filtering, a total of 1.39 billion clean reads were generated, consisting of 235.2 million, 221.9 million, and 252.4 million reads for each aestivation sample and 214.7 million, 223.2 million, and 240.5 million reads for each non-aestivation sample (Table 2). The bisulfite conversion rate (%) of all sequencing libraries ranges from 99.82% to 99.90%. After read alignment, clean reads were mapped to the reference genome of sea cucumber with mapping rates ranging from 62.38% to 63.45% (Table 2).

### 3.3. Proportion of Methylation Contexts and mCs Distribution across Genomic Features

Hierarchical clustering analysis revealed that the samples were clustered according to their groups (aestivation groups and control groups) (Supplementary Material Figure S1). PCA analysis showed that the first principal component could clearly divide samples into aestivation groups and control groups (Supplementary Material Figure S2). The methylation level distribution of *A. japonicus* genome showed the methylome’s overall characteristics (Supplementary Material Figure S3). The methylation levels of approximately 35% of all mCG were hypermethylated (methylation level >90%). However, only 10% of mCHH and mCHG were hypermethylated (methylation level >90%) compared with mCG. The methylated Cs mostly occur in the form of mCG, followed by mCHH and mCHG. The methylation level distribution of mC and mCG were alike. The methylated Cs mostly occur in the form of mCG; approximately 94% of all detected mCs (Table 3). Only 1.2% and 4% of detected mCs were mCHG and mCHH (Table 3). The proportion of mCG was significantly lower in the aestivation group than that of the control group (average proportion in control: 94.841%, average proportion in aestivation: 94.689%, *p* = 0.029). The proportion of non-CpG methylation, mCHG showed no differences between control and aestivation groups (control: 1.202%, aestivation: 1.23%, *p* = 0.105), whereas proportion of mCHH was significantly higher in the aestivation group than that in the control group (control: 3.956%, aestivation: 4.082%, *p* = 0.019).

**Figure 2:**
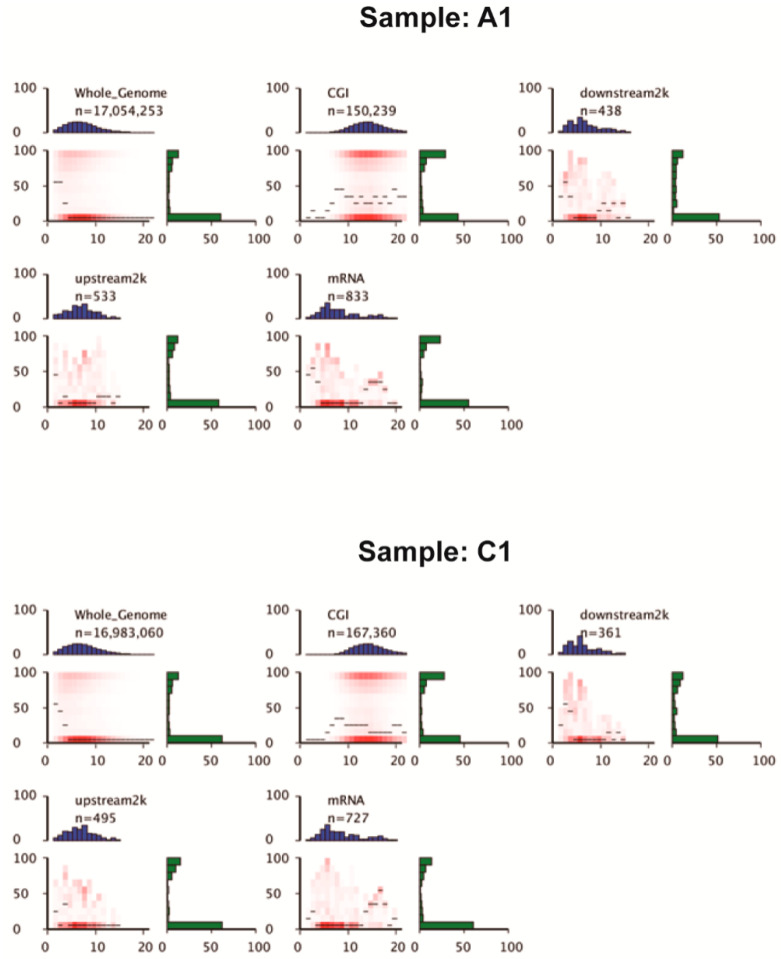
Heat maps of CpG density patterns (Sample A1 and Sample C1 as representative samples for aestivation and control groups). CpG density (*x*-axis) is defined as the number of CpG dinucleotides in 200 bp windows. The methylation level (*y*-axis) is defined as the average methylation level of cytosines in CpGs. The thin black lines denote the median methylation level of CpGs at the given local density. The red color gradient indicates an abundance of CpGs that fall into bins of given methylation levels and CpG densities. The blue bar charts show the distribution of CpG densities. The green bar charts show the distribution of methylation levels.

**Figure 3:**
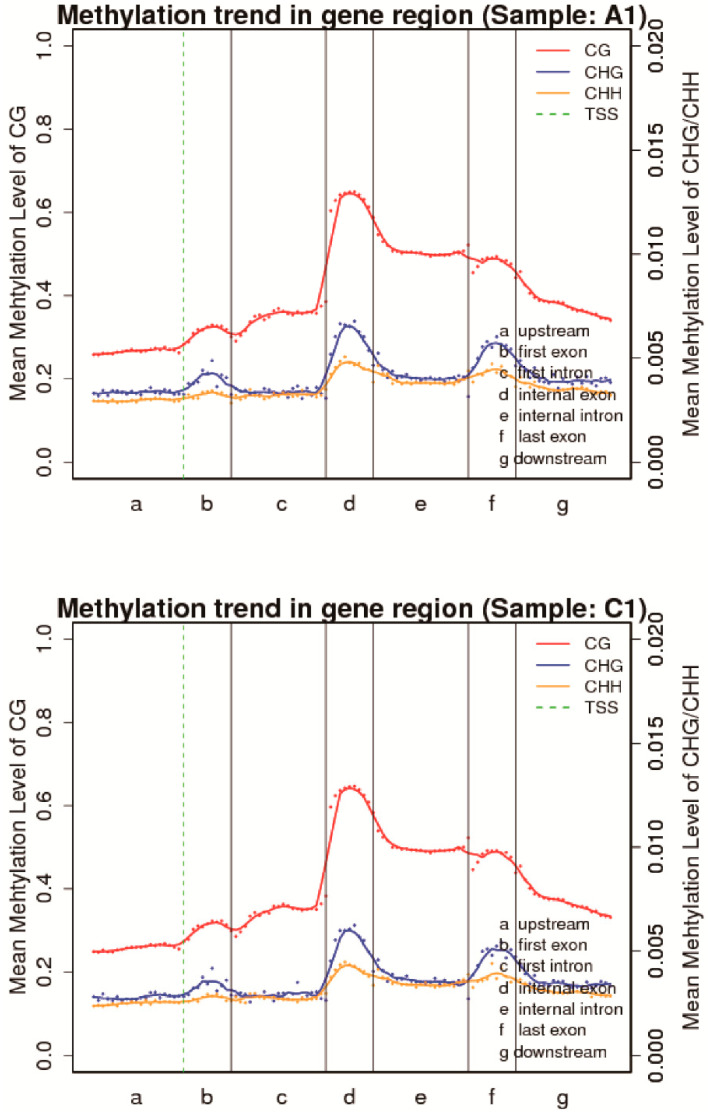
Canonical DNA methylation profiles of the entire transcriptional units (Sample A1 and Sample C1 as representative samples for aestivation and control groups). The canonical gene structure is defined by seven different features. The length of each feature was normalized and divided into equal numbers of bins. Each dot denotes the mean methylation level per bin, and the respective lines denote the 5-bin moving average. The green vertical line indicates the mean location of the transcription start sites.

The heatmap presents the methylation landscape in different genomic features (whole genome, CGI, downstream 2 kb, upstream 2 kb, mRNA) (Figure 2 and Supplementary Material Figures S4–S7). CpG islands contained the highest numbers of CpG sites (approximately 10–20 CpG sites in a 200 bp window) compared with other genomic features. About 30% of CpG sites in CpG islands were hypermethylated (methylation levels >90%) in the heatmap (Figure 2 and Supplementary Material Figure S4–S7). The other genomic features (downstream 2 kb, upstream 2 kb, mRNA) generally contained 0–10 CpG sites in the 200 bp window. A higher proportion of CpG sites within mRNA and upstream 2 kb were hypomethylated (methylation levels < 10%) than CpG sites within downstream 2 kb. The DNA methylation patterns across the entire transcriptional units (upstream, first exon, first intron, internal exon, internal exon, last exon, downstream) at the whole-genome level can help study the changes of methylation levels in distinct functional elements (Figure 3 and Supplementary Material Figure S8). Methylation differences between CG and non-CpG methylation (CHG and CHH) are visible (Figure 3 and Supplementary Material Figure S8), as methylation levels of CG are higher than those of CHG and CHH across the entire transcriptional units. Another feature is a distinct elevation in methylation level at internal exons with clear demarcation of intron/exon boundaries during transcriptional unit scanning. The lowest methylation level occurs in the first exons, followed by the last exons and the internal exons.

The average methylation levels of the whole genome, CDS, intron, and mRNA were listed in Table 4. The average methylation levels of CG, CHG, and CHH at the whole genome levels were 27.55%, 0.33%, and 0.30% in the aestivation group, and 26.53%, 0.27%, and 0.23% in the control group. The average methylation levels of CG, CHG, and CHH within CDS were 43.57%, 0.42%, and 0.50% in the aestivation group, and 44.48%, 0.39%, and 0.52% in the control group. The average methylation levels of CG, CHG, and CHH within intron were 19.26%, 0.35%, and 0.29% in the aestivation group, and 19.32%, 0.28%, and 0.24% in the control group. Within mRNA, the average methylation levels of CG, CHG, and CHH were 26.07%, 0.34%, and 0.29% in the aestivation group, and 25.35%, 0.27%, and 0.25% in the control group. The average methylation levels within CDS were the highest, suggesting that cytosine methylation primarily occurs mainly in genes. This result was consistent with other marine invertebrates, such as Pacific oysters. In oyster, CDS showed the highest methylation levels than other genomic features [35]. The average methylation of CG showed no differences between control and aestivation groups within CDS (*p* = 0.453), intron (*p* = 0.92), and mRNA (*p* = 0.33). The methylation level of CHG showed a significantly increased within intron and mRNA in the aestivation group compared with the control group (intron: *p* = 0.012; mRNA: *p* = 0.004). The methylation level of CHH showed significantly increased within intron and mRNA in the aestivation group compared with the control group (intron: *p* = 0.008; mRNA: *p* = 0.008).

### 3.4. Identification and Enrichment Analysis of Differential Methylated Regions

A total of 37,378 DMRs were identified among all detected sea cucumber samples. A total of 1393 genes were in hypermethylated DMRs, and 749 genes were in hypomethylated DMRs (Supplementary Material Tables S1 and S2). When focusing on promotors, 774 and 465 genes were associated with hypermethylated DMRs and hypomethylated DMRs (Supplementary Material Tables S3 and S4).

**Figure 4:**
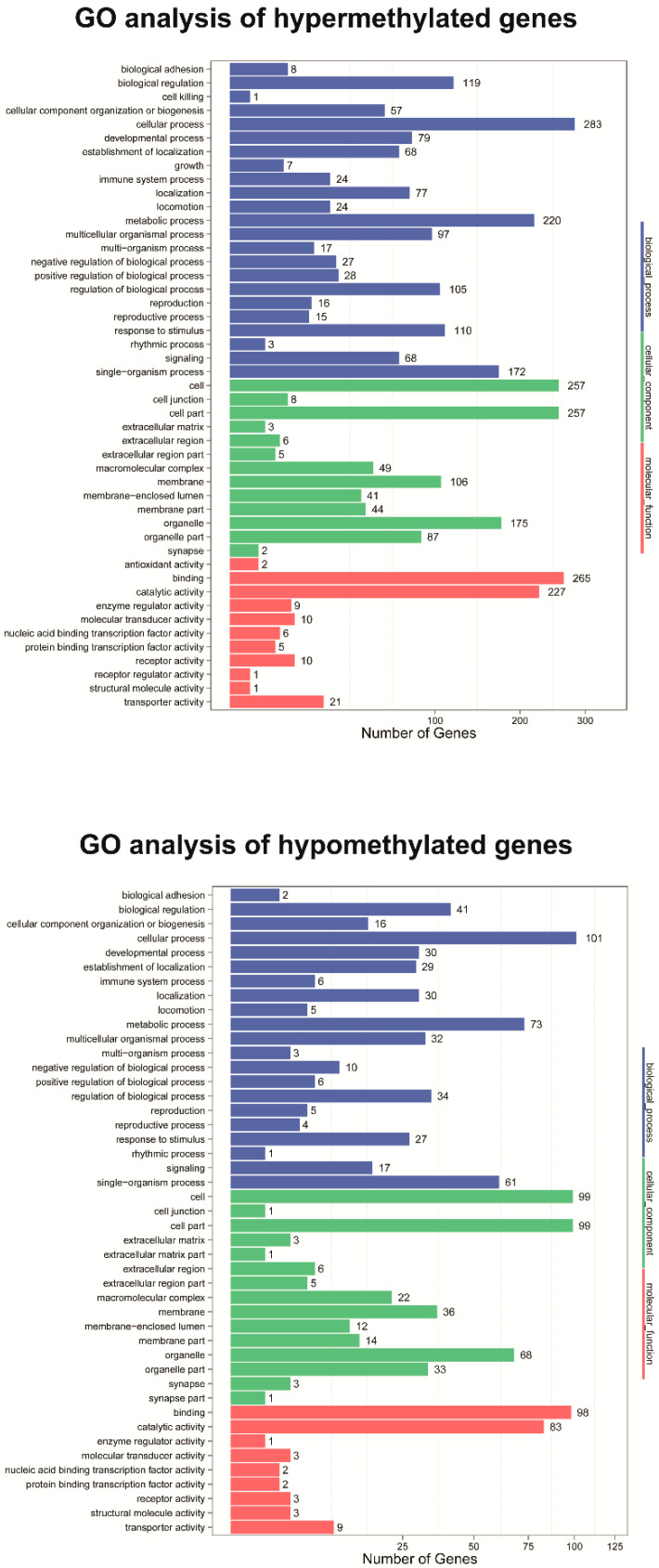
GO analysis of differentially methylated regions (DMRs)-related genes. The *x*-axis represents three domains of GO while the *y*-axis represents the gene number in every pathway and processes.

**Figure 5:**
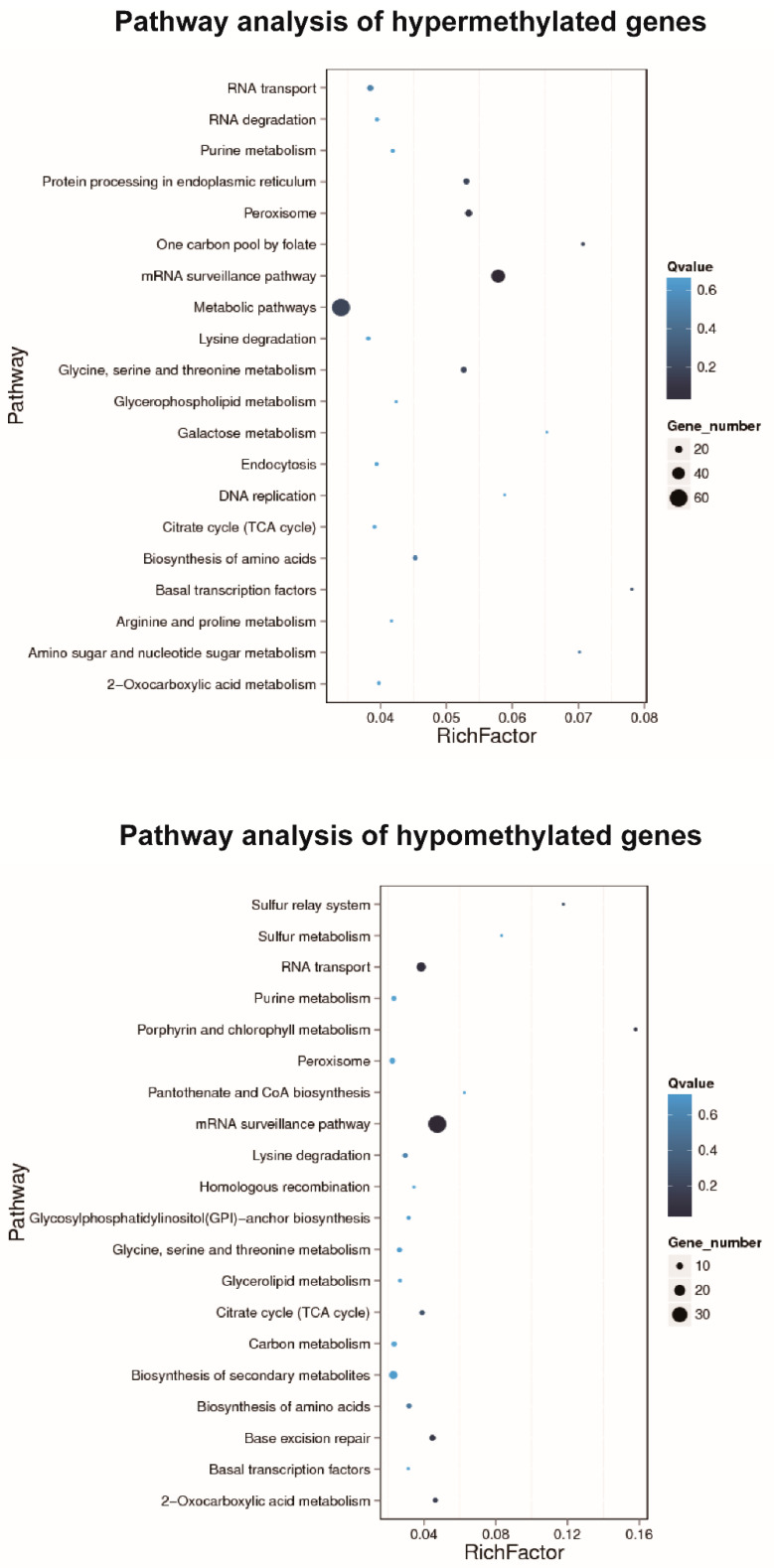
Pathway analysis of DMRs-related genes.

GO enrichment analysis of DMR-related genes provided significantly enriched GO terms corresponding to specific molecular function, cellular component, and biological process (Figure 4). The top enriched GO terms in the biological process are cellular process, metabolic process, and single-organism processes. The over-represented GO terms in the cellular component are cell, cell part, and organelle. In terms of molecular function, the top enriched GO terms are binding, catalytic activity, and transporter activity. KEGG pathway enrichment analysis indicated that the annotated genes within hypermethylated DMRs were enriched in metabolic pathways and the mRNA surveillance pathway (Figure 5). In contrast, the annotated genes within hypomethylated DMRs were enriched in the mRNA surveillance pathway and RNA transport (Figure 5).

A total of 44 hypermethylated genes and 29 hypomethylated genes were involved in the mRNA surveillance pathway (Supplementary Material Tables S3 and S4). Among genes associated with mRNA surveillance, many genes were RPPT genes (Retrovirus-related Pol polyprotein from transposon). A total of 24 hypermethylated and 15 hypomethylated RPPT genes were found involved in mRNA surveillance. Enriched differentially methylated genes associated with the mRNA surveillance pathway included Retrotransposable element Tf2 155 kDa protein (type 1 and type 3), Transposon Ty3-I Gag-Pol polyprotein, and Pro-Pol polyprotein. Interestingly, many other members of RPPT genes were also found to be differentially methylated. A total of 37 RPPT genes were in the hypermethylated gene list and 26 RPPT genes in the hypomethylated gene list. Promotors of 42 RPPT genes and 29 RPPT genes were associated with hypermethylated DMRs and hypomethylated DMRs.

## 4. Discussion

An increasing number of studies reveal that epigenetic controls contribute to the differential gene expression during environmentally induced hypometabolism and seasonal change [12,36,37,38,39]. In *A. japonicus*, the transcriptional changes of methylation associated enzymes DNMT1 and MBD2/3 were up-regulated during aestivation [40,41]. In our study, DNA methylation modifiers, including DNA methyltransferases (DNMT1, DNMT3a), methyl-CpG-binding domain proteins (MBD2/3, MBD4, MBD5, MBD6), DNA demethylases (TET, TDG) were all up-regulated in aestivating groups (Figure 1). In thirteen-lined ground squirrels, the altered expression of MBDs and MECP2 (methyl CpG binding protein 2) expression levels accompanies different stages of hibernation [42]. In read-eared slider turtle, an overall increase in DNMT protein expression in liver and muscle was observed during anoxia-induced hypometabolism [43]. The significant changes in methylation-related enzymes indicate that DNA methylation machinery contributes to regulatory response to environmental induced hypometabolism.

In aquatic species, there is still limited research suggesting that DNA methylation has regulatory functions in genes involved in environmental-induced aestivation. In the present study, we reported a genome-wide examination of DNA methylation in the intestine of sea cucumber *A. japonicus* during thermal stress-induced hypometabolism. The mapping rates of clean reads approximately ranged from 62.38% to 63.45% (Table 2), which is above the average value reported in other species that bisulfite-converted DNA typically has a mapping efficiency closer to 30–50% [44]. However, the WGBS results obtained from this study are also consistent because bisulfite conversion reduces the complexity of the genomic sequence and reduces the ability of most computational programs to align sequences onto the reference genome.

Further DNA methylation profile analysis was also conducted in our present study across the distinct genomic features and entire transcriptional units. Interestingly, a significant increase in non-CpG methylation (CHG and CHH) was observed within the intron and mRNA regions in aestivation groups. This result suggested that non-CpG methylation may be more vital to regulate aestivation in *A. japonicus* than CpG methylation. A whole-genome methylation landscape of constant heat-stressed pigs showed similar proportions of methylation at CpG sites but showed significantly decreased percentages at non-CpG sites with control pigs [45]. Non-CpG methylation is controlled through DNMT3a [46] and DNMT3b [47]. DNMT3a was highly up-regulated in the aestivation groups, potentially contributing to higher non-CpG methylation levels. A distinct elevation in methylation level at internal exons was observed with clear demarcation of intron/exon boundaries during transcriptional unit scanning. The lowest methylation level occurs in the first exons, followed by the last exons and the internal exons. This result was consistent with previous findings in other marine invertebrates [48], suggesting that the first exons appear more prone to the mutagenic effects. In contrast, internal exons are more influenced by the regulatory impact of DNA methylation and may play a role in constructing alternative splicing.

To better understand the functional classification of DMRs during aestivation, GO enrichment analysis was conducted to compare aestivation groups and control groups. The top enriched GO terms of DMRs identified in the present study were mainly associated with the term “cellular process” and “metabolic process” which suggests that DNA methylation may have an essential role in regulating global transcription during aestivation-induced hypometabolism. The enriched pathway analysis played a crucial role in identifying the pathways most strongly affected by DNA methylation. The results indicated that the annotated genes within hypermethylated DMRs were enriched in metabolic pathways and the mRNA surveillance pathway. As aestivation led to a low metabolic rate, energy redistribution, and defense mechanisms enhancement [19], the expression patterns of many genes associated with metabolic pathways might be altered. The annotated genes within hypomethylated DMRs were enriched in the mRNA surveillance pathway and RNA transport. The mRNA surveillance pathway is a mechanism that ensures the quality of mRNAs and detects translational errors and degrades abnormal mRNAs [49]. The mRNA surveillance pathway is also required for oxidative stress tolerance, which inhibits the generation of abnormal proteins from translation error, NSD (non-stop decay) [50]. Another recent study revealed that an mRNA surveillance system contributed to *E. coli* cold shock adaptation program [51]. Therefore, the mRNA surveillance pathway might be one of the essential adaptive strategies through DNA methylation modification for aestivating sea cucumbers (*A. japonicus*).

KEGG enrichment analysis of the genes with significant methylation difference indicates that these genes were mostly enriched in the mRNA surveillance pathway. Interestingly, mRNA surveillance pathway was mainly composed of retrovirus-related Pol polyprotein from transposon (RPPT) genes, one of the most significantly expanded gene families in *A. japonicus* genome that predominately accumulated in LG01 [28]. A total of 24 hypermethylated and 15 hypomethylated retrovirus-related pol polyprotein from transposon (RPPT) genes were found involved in mRNA surveillance, which might be potentially important regulators for aestivation. These genes are members of retrotransposons, which regulate transcription levels of their adjacent genes by genes coming under the control of their promoter [52,53].

The regulation of retrovirus-related transposon has been observed in many other species in response to environmentally induced stress. The expression of RPPT 412 was up-regulated when snails suffered thermal stress [54]. RPPT genes, including RPPT 17.6, RPPT 297, and RPPT 412, were significantly up-regulated in response to early heat stress in corals [55]. Enriched differentially methylated genes associated with the mRNA surveillance pathway also included transposon Ty3-I Gag-Pol polyprotein and Pro-Pol polyprotein. Gag-Pol polyprotein genes were detected and regulated when exposed to heat and cold stresses in manila clam [56]. These studies all support our current findings.

Retrotransposons are characterized by typical long terminal repeats (LTRs), which can amplify themselves and insert them into the genome at target sites [57]. The presence of active promoters within retrotransposons, because of reduced DNA methylation, can affect the standard transcription, resulting in reduced mRNA levels or translation capacity, or disrupted transcription initiation [58]. Additionally, retrotransposons could alter the genetic information and restructure genomes through chromosomal rearrangements [59,60]. The transposition rates of retrotransposons could contribute to genome evolution [61]. Retrovirus-related Pol polyprotein from transposon genes are among the significantly expanded gene families in sea cucumber [28]. Adaptive expansion of the RPPT gene family in the *A. japonicus* genome might be a more efficient regulatory mechanism on global gene expression during aestivation through differential methylation.

## 5. Conclusions

Our findings provide a comprehensive, detailed picture of DNA methylation patterns in *A. japonicus* during environmental-induced aestivation. Most of the methylation-related enzymes were up-regulated during aestivation. Whole methylome analysis suggested that DNA methylation may have an essential role in regulating global transcription during environmental induced hypometabolism. Further DNA methylation profile analysis was also conducted across the distinct genomic features and entire transcriptional units. A significant increase in non-CpG methylation (CHG and CHH) was observed within the intron and mRNA regions in aestivation groups. A total of 1393 genes were in hypermethylated DMRs, and 749 genes were in hypomethylated DMRs. Differentially methylated genes were enriched in the mRNA surveillance pathway, metabolic pathway, and RNA transport. Many retrovirus-related transposon genes modified by DNA methylation might be potentially essential regulators for aestivation. This study provides further understanding of epigenetic control on environmental induced hypometabolism in aquatic organisms. However, explicit confirmation of molecular mechanisms requires future intensive research, which may eventually aid in understanding the complexity of marine species’ temperature-induced aestivation mechanisms.

## Figures and Tables

**Table 1:** Primer sequences for real-time qPCR.

Names	Sequences
Dnmt1	F: ATATGGCGTCCCACAGACTC
	R: CACAGTGATGGTGCGATAGG
Dnmt3a	F: CAGACGCGATCAAAGTCTCA
	R: CCCCAATCAACAGGTCAAAT
Dnmt3b	F: CGTCGTTACAGAGGGGAGAG
	R: ACCACTTTCAGGGAGGGACT
MBD2/3	F: CTTTGACCTTAGCGCCTTTG
	R: GTTCTGGGCGTGATTTGTTT
MBD4	F: ACCAGTCCGAATCAACCAAC
	R: AACCAATCCTCGTCAGTTCC
MBD5	F: ACGGAGATGTCAAAGGGCAG
	R: GCTGCCTCCAAGTTAGTCGT
MBD6	F: ATCGTCTCCTGCCTCCTCTT
	R: GATGCTGCTGTGCTTCGATG
TDG	F: CGTTATGCCTTCGTCAAGTG
	R: ATTCACCGCCTTCAGTTCAT
TET2	F: ACCAGCAGAGGAAGGGAAAT
	R: TGGAGGCATACGAGGCTACT

**Table 2:** Data summary and alignment statistics with reference genome.

Sample ID	Clean Reads	Clean Data Size(bp)	Mapped Reads	Mapping Rate (%)	Uniquely Mapped Reads	Uniquely Mapping Rate (%)	Bisulfite Conversion Rate (%)
A1	235,240,966	29,405,120,750	146,748,774	62.38	120,561,967	51.25	99.83
A2	221,903,100	27,737,887,500	138,682,431	62.50	114,085,412	51.41	99.82
A3	252,389,374	31,548,671,750	158,828,900	62.93	130,699,039	51.78	99.83
C1	214,743,374	26,842,921,750	134,186,828	62.49	109,061,960	50.79	99.87
C2	223,167,750	27,895,968,750	141,594,447	63.45	114,184,023	51.17	99.90
C3	240,490,792	30,061,349,000	151,604,875	63.04	124,088,420	51.60	99.89

**Table 3:** Proportion of CG, CHG and CHH in all methyl-cytosine.

Sample		mCG	mCHG	mCHH
A1	mC number	6,190,426	80,195	266,577
	Proportion (%)	94.695	1.227	4.078
A2	mC number	5,984,027	77,057	256,981
	Proportion (%)	94.713	1.22	4.067
A3	mC number	6,385,314	83,819	276,569
	Proportion (%)	94.658	1.243	4.1
C1	mC number	6,017,278	76,797	253,064
	Proportion (%)	94.803	1.21	3.987
C2	mC number	6,111,110	78,486	257,136
	Proportion (%)	94.794	1.217	3.989
C3	mC number	5,752,504	71,494	235,918
	Proportion (%)	94.927	1.18	3.893

**Table 4:** Average methylation levels of different genomic regions.

Group	Regions	C (%)	CG (%)	CHG (%)	CHH (%)
Aestivation Group 1	genome	3.564	28.113	0.331	0.293
	CDS	7.079	43.595	0.478	0.487
	intron	2.649	20.231	0.341	0.295
	mRNA	3.463	26.968	0.342	0.291
Aestivation Group 2	genome	3.476	27.337	0.333	0.298
	CDS	6.909	42.708	0.396	0.526
	intron	2.433	18.562	0.346	0.285
	mRNA	3.257	25.355	0.34	0.291
Aestivation Group 3	genome	3.429	27.199	0.329	0.294
	CDS	7.022	44.404	0.374	0.472
	intron	2.467	18.98	0.355	0.293
	mRNA	3.316	25.896	0.338	0.295
Control Group 1	genome	3.521	27.1	0.281	0.25
	CDS	7.479	46.443	0.408	0.491
	intron	2.602	19.658	0.298	0.256
	mRNA	3.414	26.121	0.289	0.258
Control Group 2	genome	3.329	24.957	0.248	0.217
	CDS	6.966	43.446	0.331	0.455
	intron	2.476	18.712	0.248	0.219
	mRNA	3.255	24.56	0.246	0.232
Control Group 3	genome	3.529	27.534	0.269	0.235
	CDS	7.192	43.552	0.432	0.627
	intron	2.62	19.591	0.288	0.232
	mRNA	3.342	25.364	0.268	0.258

## Supplementary Materials

The following are available online at https://www.mdpi.com/2073-4425/11/9/1020/s1, Figure S1: Cluster analysis of similarities among methylation profiles of aestivation groups and control groups; Figure S2: Sample structure identified by principal component analysis (PCA) with the first 2 principal components; Figure S3: Methylation level distribution of mC; Figure S4: Heat maps show CpG density patterns of A2 sample; Figure S5: Heat maps show CpG density patterns of A3 sample; Figure S6: Heat maps show CpG density patterns of C2 sample; Figure S7: Heat maps show CpG density patterns of C3 sample; Figure S8: Canonical DNA methylation profiles of the entire transcriptional units; Table S1: Gene list annotated within hypermethylated DMRs; Table S2: Gene list annotated within hypomethylated DMRs; Table S3: Hypermethylated gene list involved in the mRNA surveillance pathway; Table S4: Hypomethylated gene list involved in the mRNA surveillance pathway.

## References

[1] Price T.D., Qvarnström A., Irwin D.E. (2003). The role of phenotypic plasticity in driving genetic evolution. Proc. R. Soc. Lond. B Biol. Sci..

[2] Heldmaier G., Ortmann S., Elvert R. (2004). Natural hypometabolism during hibernation and daily torpor in mammals. Respir. Physiol. Neurobiol..

[3] Storey K.B., Storey J.M. (2012). Aestivation: Signaling and hypometabolism. J. Exp. Biol..

[4] Renfree M.B., Shaw G. (2000). Diapause. Annu. Rev. Physiol..

[5] van Breukelen F., Martin S.L. (2015). The hibernation continuum: Physiological and molecular aspects of metabolic plasticity in mammals. Physiology.

[6] Canale C.I., Henry P.-Y. (2010). Adaptive phenotypic plasticity and resilience of vertebrates to increasing climatic unpredictability. Clim. Res..

[7] Winterhalter W.E., Mousseau T.A. (2007). Patterns of phenotypic and genetic variation for the plasticity of diapause incidence. Evol. Int. J. Org. Evol..

[8] Wolffe A.P., Matzke M.A. (1999). Epigenetics: Regulation through repression. Science.

[9] Goldberg A.D., Allis C.D., Bernstein E. (2007). Epigenetics: A landscape takes shape. Cell.

[10] Kronholm I., Collins S. (2016). Epigenetic mutations can both help and hinder adaptive evolution. Mol. Ecol..

[11] Wilschut R.A., Oplaat C., Snoek L.B., Kirschner J., Verhoeven K.J. (2016). Natural epigenetic variation contributes to heritable flowering divergence in a widespread asexual dandelion lineage. Mol. Ecol..

[12] Morin P., Storey K.B. (2009). Mammalian hibernation: Differential gene expression and novel application of epigenetic controls. Int. J. Dev. Biol..

[13] Storey K.B. (2015). Regulation of hypometabolism: Insights into epigenetic controls. J. Exp. Biol..

[14] Tessier S.N., Luu B.E., Smith J.C., Storey K.B. (2017). The role of global histone post-translational modifications during mammalian hibernation. Cryobiology.

[15] Jeremias G., Barbosa J., Marques S.M., Asselman J., Gonçalves F.J., Pereira J.L. (2018). Synthesizing the role of epigenetics in the response and adaptation of species to climate change in freshwater ecosystems. Mol. Ecol..

[16] Roberts S.B., Gavery M.R. (2012). Is there a relationship between DNA methylation and phenotypic plasticity in invertebrates?. Front. Physiol..

[17] Gavery M.R., Roberts S.B. (2010). DNA methylation patterns provide insight into epigenetic regulation in the Pacific oyster (*Crassostrea gigas*). BMC Genom..

[18] Putnam H.M., Davidson J.M., Gates R.D. (2016). Ocean acidification influences host DNA methylation and phenotypic plasticity in environmentally susceptible corals. Evol. Appl..

[19] Storey K.B., Storey J.M. (2010). Metabolic Regulation and Gene Expression during Aestivation. Aestivation.

[20] Wang F., Yang H., Gao F., Liu G. (2008). Effects of acute temperature or salinity stress on the immune response in sea cucumber, Apostichopus japonicus. Comp. Biochem. Physiol. Part A Mol. Integr. Physiol..

[21] Yang H., Hamel J.-F., Mercier A. (2015). The Sea Cucumber Apostichopus Japonicus: History, Biology and Aquaculture.

[22] Gao F., Yang H., Xu Q., Wang F., Liu G., German D.P. (2008). Phenotypic plasticity of gut structure and function during periods of inactivity in Apostichopus japonicus. Comp. Biochem. Physiol. Part B Biochem. Mol. Biol..

[23] Zhu A., Chen M., Zhang X., Storey K.B. (2016). Gene structure, expression, and DNA methylation characteristics of sea cucumber cyclin B gene during aestivation. Gene.

[24] Li Y., Wang R., Xun X., Wang J., Bao L., Thimmappa R., Ding J., Jiang J., Zhang L., Li T. (2018). Sea cucumber genome provides insights into saponin biosynthesis and aestivation regulation. Cell Discov..

[25] Gao F., Yang H., Xu Q., Wang F., Liu G. (2009). Effect of water temperature on digestive enzyme activity and gut mass in sea cucumber Apostichopus japonicus (Selenka), with special reference to aestivation. Chin. J. Oceanol. Limnol..

[26] Zhao Y., Yang H., Storey K.B., Chen M. (2014). RNA-seq dependent transcriptional analysis unveils gene expression profile in the intestine of sea cucumber Apostichopus japonicus during aestivation. Comp. Biochem. Physiol. Part D Genom. Proteom..

[27] Zhao Y., Chen M., Wang T., Sun L., Xu D., Yang H. (2014). Selection of reference genes for qRT-PCR analysis of gene expression in sea cucumber Apostichopus japonicus during aestivation. Chin. J. Oceanol. Limnol..

[28] Zhang X., Sun L., Yuan J., Sun Y., Gao Y., Zhang L., Li S., Dai H., Hamel J.-F., Liu C. (2017). The sea cucumber genome provides insights into morphological evolution and visceral regeneration. PLoS Biol..

[29] Xi Y., Li W. (2009). BSMAP: Whole genome bisulfite sequence MAPping program. BMC Bioinform..

[30] Akalin A., Kormaksson M., Li S., Garrett-Bakelman F.E., Figueroa M.E., Melnick A., Mason C.E. (2012). methylKit: A comprehensive R package for the analysis of genome-wide DNA methylation profiles. Genome Biol..

[31] Xiang H., Zhu J., Chen Q., Dai F., Li X., Li M., Zhang H., Zhang G., Li D., Dong Y. (2010). Single base–resolution methylome of the silkworm reveals a sparse epigenomic map. Nat. Biotechnol..

[32] Lister R., Pelizzola M., Dowen R.H., Hawkins R.D., Hon G., Tonti-Filippini J., Nery J.R., Lee L., Ye Z., Ngo Q.-M. (2009). Human DNA methylomes at base resolution show widespread epigenomic differences. Nature.

[33] Li Y., Zhu J., Tian G., Li N., Li Q., Ye M., Zheng H., Yu J., Wu H., Sun J. (2010). The DNA methylome of human peripheral blood mononuclear cells. PLoS Biol..

[34] Kanehisa M., Goto S. (2000). KEGG: Kyoto encyclopedia of genes and genomes. Nucleic Acids Res..

[35] Wang X., Li Q., Lian J., Li L., Jin L., Cai H., Xu F., Qi H., Zhang L., Wu F. (2014). Genome-wide and single-base resolution DNA methylomes of the Pacific oyster Crassostrea gigas provide insight into the evolution of invertebrate CpG methylation. BMC Genom..

[36] Hudson N.J., Lonhienne T., Franklin C.E., Harper G.S., Lehnert S. (2008). Epigenetic silencers are enriched in dormant desert frog muscle. J. Comp. Physiol. B.

[37] Krivoruchko A., Storey K.B. (2010). Epigenetics in anoxia tolerance: A role for histone deacetylases. Mol. Cell. Biochem..

[38] Seibel B.A., Häfker N.S., Trübenbach K., Zhang J., Tessier S.N., Pörtner H.-O., Rosa R., Storey K.B. (2014). Metabolic suppression during protracted exposure to hypoxia in the jumbo squid, Dosidicus gigas, living in an oxygen minimum zone. J. Exp. Biol..

[39] Wijenayake S., Hawkins L.J., Storey K.B. (2018). Dynamic regulation of six histone H3 lysine (K) methyltransferases in response to prolonged anoxia exposure in a freshwater turtle. Gene.

[40] Wang T., Yang H., Zhao H., Chen M., Wang B. (2011). Transcriptional changes in epigenetic modifiers associated with gene silencing in the intestine of the sea cucumber, Apostichopus japonicus (Selenka), during aestivation. Chin. J. Oceanol. Limnol..

[41] Zhao Y., Chen M., Su L., Wang T., Liu S., Yang H. (2013). Molecular cloning and expression-profile analysis of sea cucumber DNA (Cytosine-5)-methyltransferase 1 and methyl-CpG binding domain type 2/3 genes during aestivation. Comp. Biochem. Physiol. Part B Biochem. Mol. Biol..

[42] Alvarado S., Mak T., Liu S., Storey K.B., Szyf M. (2015). Dynamic changes in global and gene-specific DNA methylation during hibernation in adult thirteen-lined ground squirrels, Ictidomys tridecemlineatus. J. Exp. Biol..

[43] Wijenayake S., Storey K.B. (2016). The role of DNA methylation during anoxia tolerance in a freshwater turtle (Trachemys scripta elegans). J. Comp. Physiol. B.

[44] Tran H., Porter J., Sun M.-A., Xie H., Zhang L. (2014). Objective and comprehensive evaluation of bisulfite short read mapping tools. Adv. Bioinform..

[45] Hao Y., Cui Y., Gu X. (2016). Genome-wide DNA methylation profiles changes associated with constant heat stress in pigs as measured by bisulfite sequencing. Sci. Rep..

[46] Ramsahoye B.H., Biniszkiewicz D., Lyko F., Clark V., Bird A.P., Jaenisch R. (2000). Non-CpG methylation is prevalent in embryonic stem cells and may be mediated by DNA methyltransferase 3a. Proc. Natl. Acad. Sci. USA.

[47] Arand J., Spieler D., Karius T., Branco M.R., Meilinger D., Meissner A., Jenuwein T., Xu G., Leonhardt H., Wolf V. (2012). In vivo control of CpG and non-CpG DNA methylation by DNA methyltransferases. PLoS Genet..

[48] Song K., Li L., Zhang G. (2017). The association between DNA methylation and exon expression in the Pacific oyster Crassostrea gigas. PLoS ONE.

[49] Isken O., Maquat L.E. (2007). Quality control of eukaryotic mRNA: Safeguarding cells from abnormal mRNA function. Genes Dev..

[50] Jamar N.H., Kritsiligkou P., Grant C.M. (2017). The non-stop decay mRNA surveillance pathway is required for oxidative stress tolerance. Nucleic Acids Res..

[51] Zhang Y., Burkhardt D.H., Rouskin S., Li G.-W., Weissman J.S., Gross C.A. (2018). A stress response that monitors and regulates mRNA structure is central to cold shock adaptation. Mol. Cell.

[52] Kim A., Terzian C., Santamaria P., Pelisson A., Purd'homme N., Bucheton A. (1994). Retroviruses in invertebrates: The gypsy retrotransposon is apparently an infectious retrovirus of Drosophila melanogaster. Proc. Natl. Acad. Sci. USA.

[53] Whitelaw E., Martin D.I. (2001). Retrotransposons as epigenetic mediators of phenotypic variation in mammals. Nat. Genet..

[54] Chu N.D., Miller L.P., Kaluziak S.T., Trussell G.C., Vollmer S.V. (2014). Thermal stress and predation risk trigger distinct transcriptomic responses in the intertidal snail Nucella lapillus. Mol. Ecol..

[55] Traylor-Knowles N., Rose N.H., Sheets E.A., Palumbi S.R. (2017). Early transcriptional responses during heat stress in the coral Acropora hyacinthus. Biol. Bull..

[56] Menike U., Lee Y., Oh C., Wickramaarachchi W., Premachandra H., Park S.C., Lee J., De Zoysa M. (2014). Oligo-microarray analysis and identification of stress-immune response genes from manila clam (Ruditapes philippinarum) exposure to heat and cold stresses. Mol. Biol. Rep..

[57] Cordaux R., Batzer M.A. (2009). The impact of retrotransposons on human genome evolution. Nat. Rev. Genet..

[58] Robertson K.D., Wolffe A.P. (2000). DNA methylation in health and disease. Nat. Rev. Genet..

[59] Wessler S.R. (2006). Transposable elements and the evolution of eukaryotic genomes. Proc. Natl. Acad. Sci. USA.

[60] Sotero-Caio C.G., Platt R.N., Suh A., Ray D.A. (2017). Evolution and diversity of transposable elements in vertebrate genomes. Genome Biol. Evol..

[61] Mita P., Boeke J.D. (2016). How retrotransposons shape genome regulation. Curr. Opin. Genet. Dev..

