# Supplementary material for: Genome-Wide DNA Methylation Signatures of Sea Cucumber Apostichopus japonicus during Environmental Induced Aestivation
Source: Genes (Basel). 2020 Aug 31;11(9):1020. doi: 10.3390/genes11091020 (PMC7565549; doi:10.3390/genes11091020)

**Supplemental Figure S1.** Cluster analysis of similarities among methylation profiles of aestivation groups and control groups.


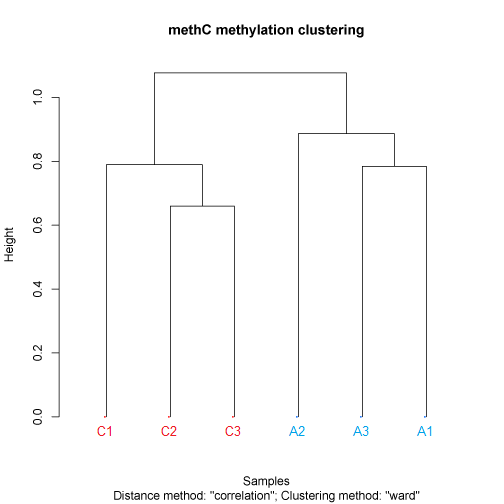


**Supplemental Figure S2.** Sample structure identified by principal component analysis (PCA) with the first 2 principal components. The coordinates are the 1st 2 principal components.


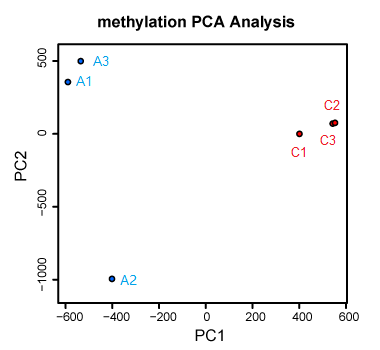


**Supplemental Figure S3.** Methylation level distribution of mC. The x-axis represented the percentage of reads showing mC at a reference site. The y-axis represented the fraction of total mC within bins of 10%.


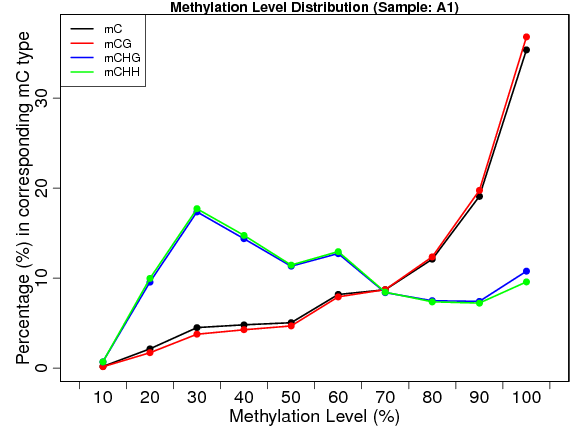

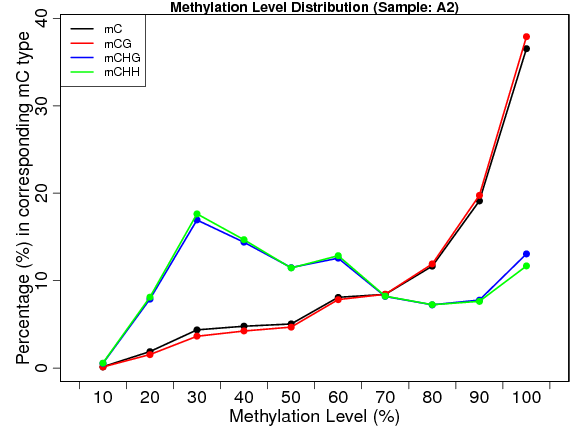


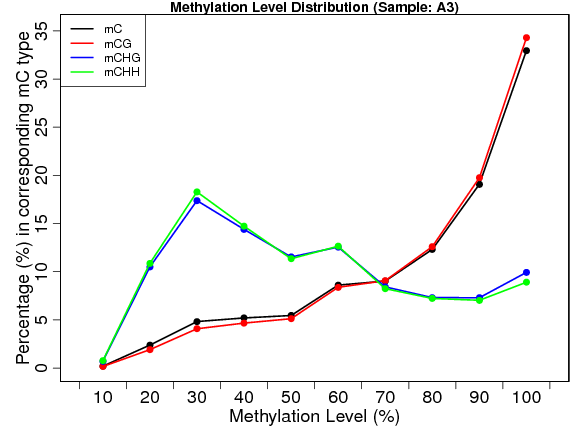


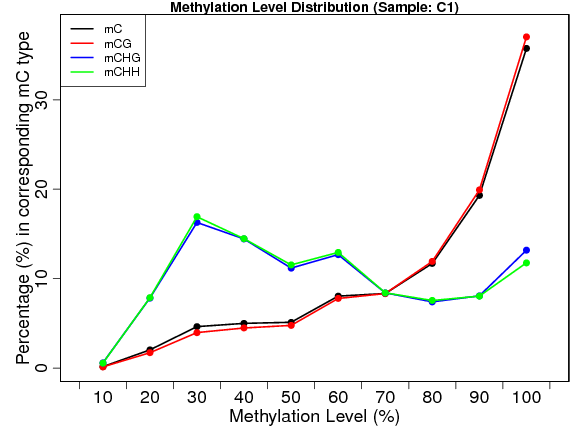


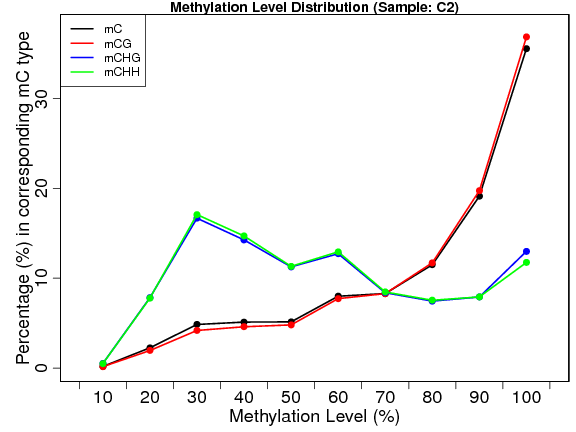

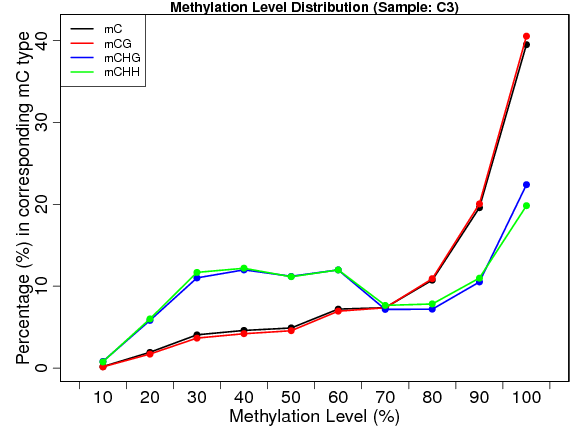


**Supplemental Figure S4.** Heat maps show CpG density patterns of A2 sample.


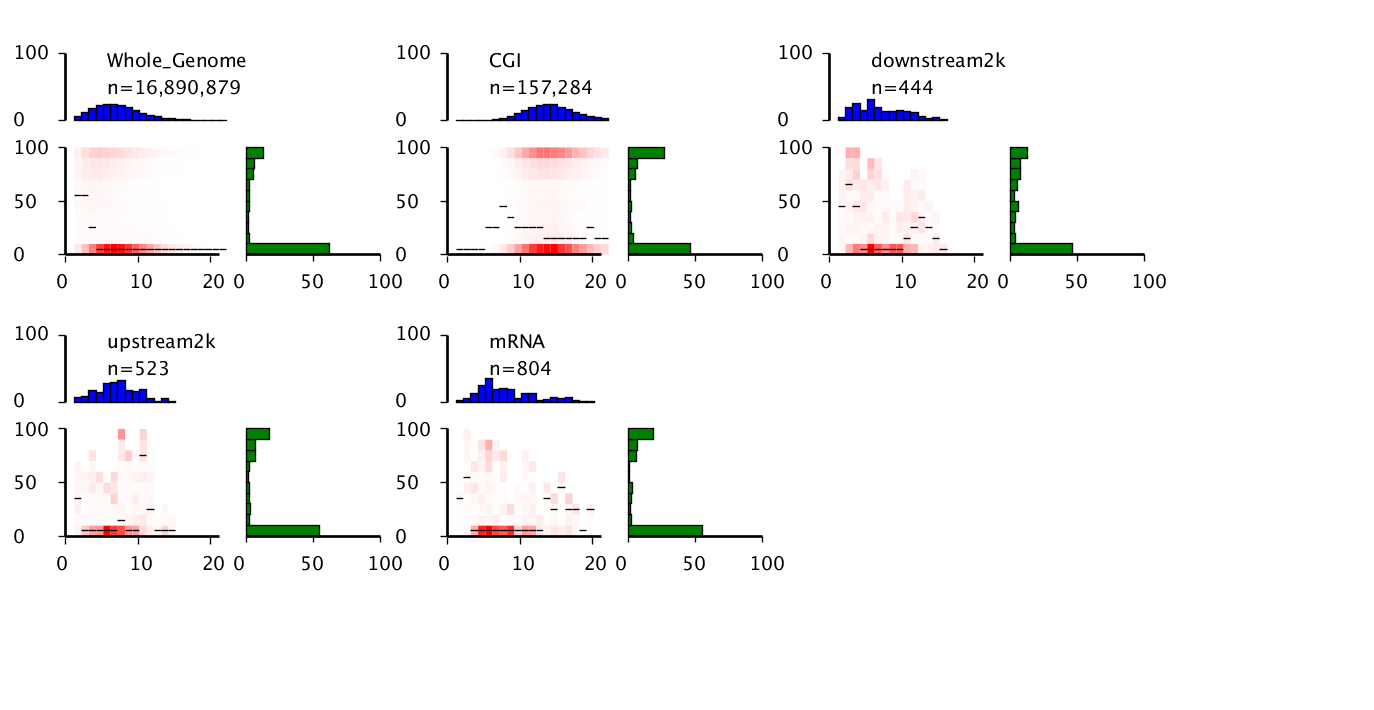


**Supplemental Figure S5.** Heat maps show CpG density patterns of A3 sample.


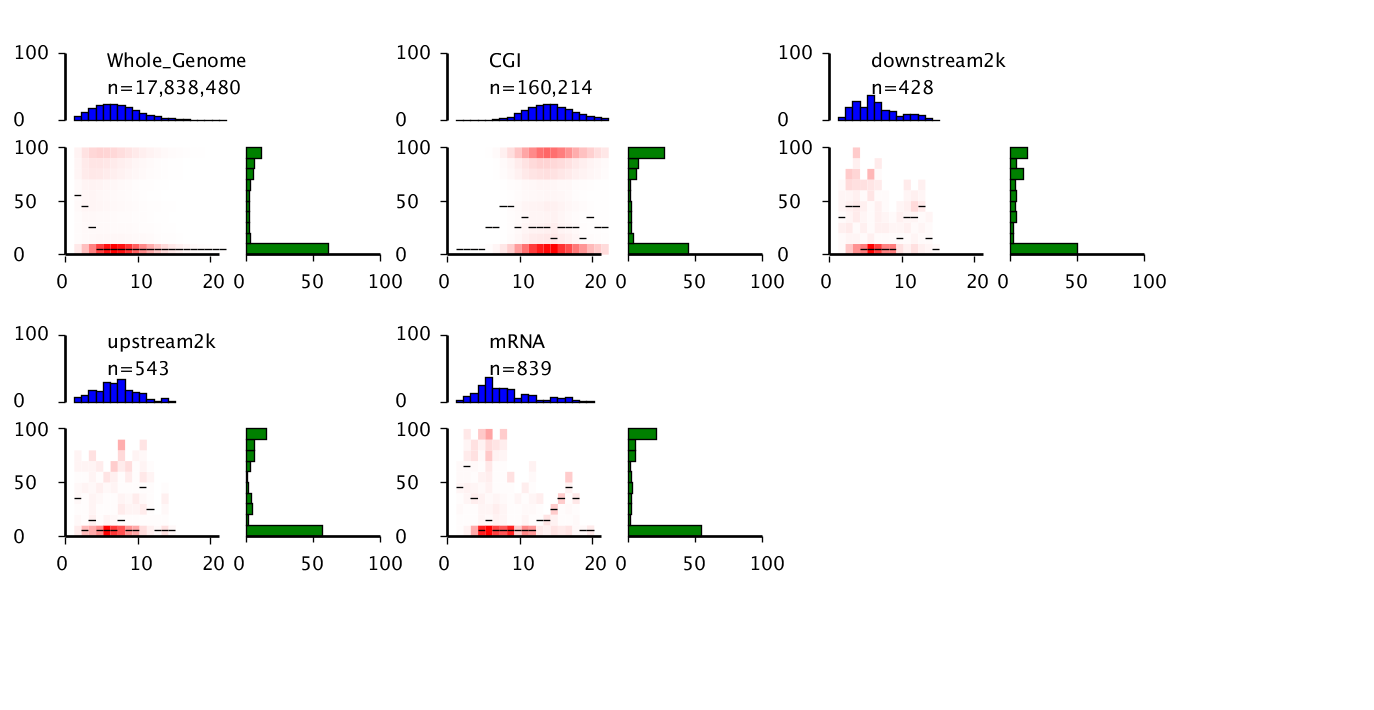


**Supplemental Figure S6.** Heat maps show CpG density patterns of C2 sample.


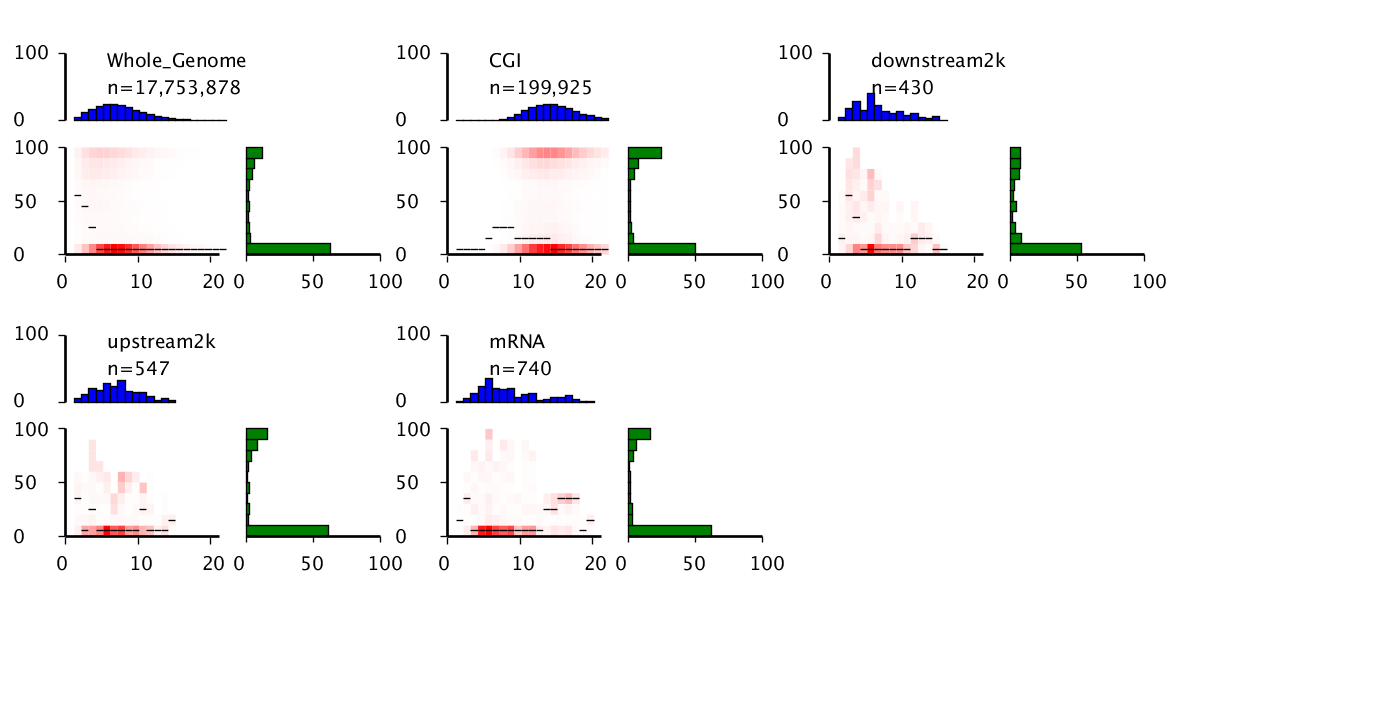


**Supplemental Figure S7.** Heat maps show CpG density patterns of C3 sample.


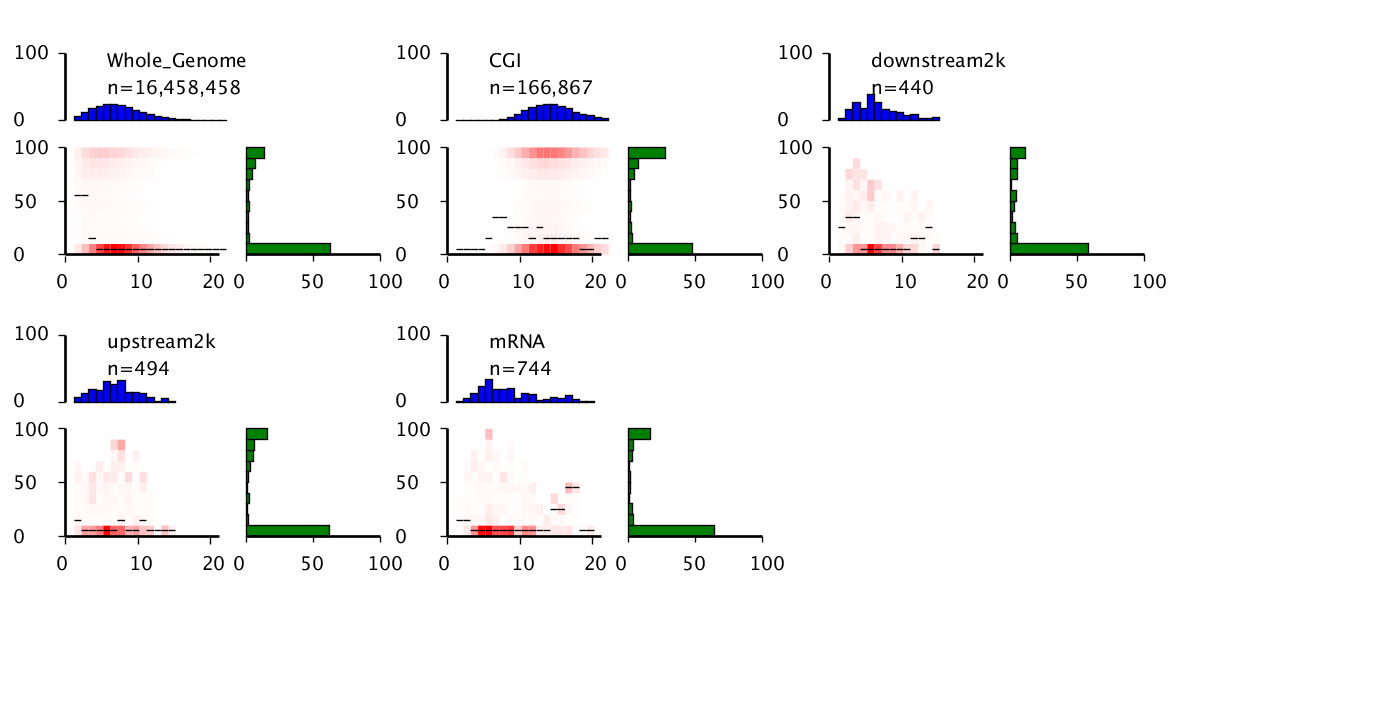


**Supplemental Figure S8.** Canonical DNA methylation profiles of the entire transcriptional units. The canonical gene structure is defined by seven different features. The length of each feature was normalized and divided into equal numbers of bins. Each dot denotes the mean methylation level per bin and the respective lines denote the 5-bin moving average. The green vertical line indicates the mean location of the transcription start sites.


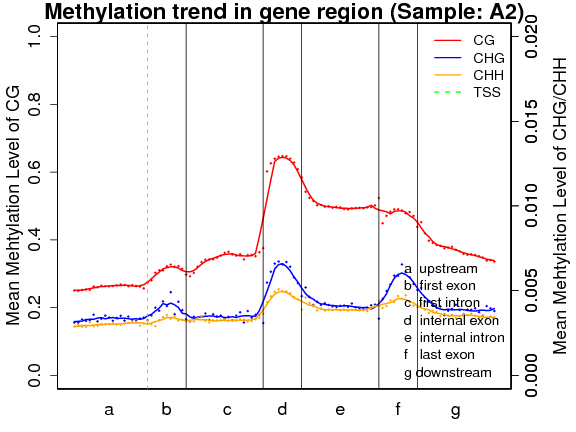


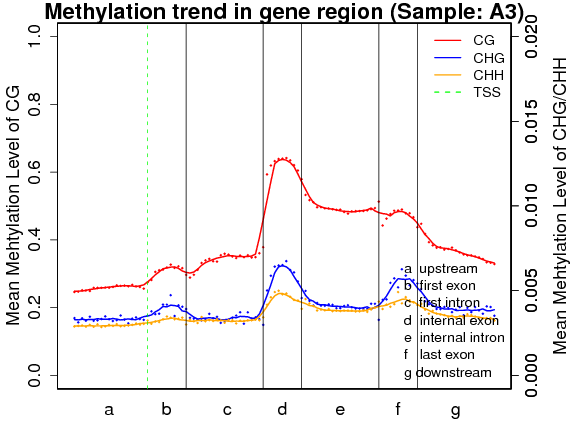


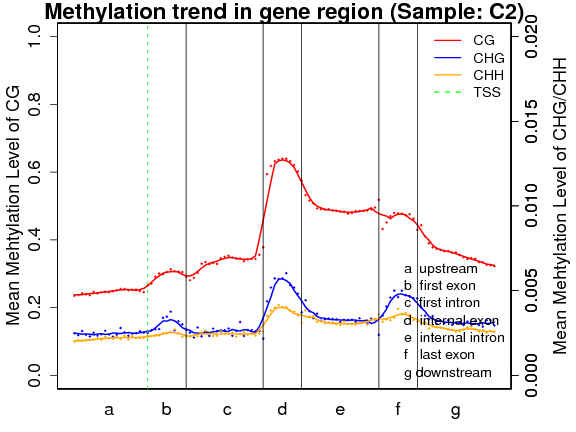


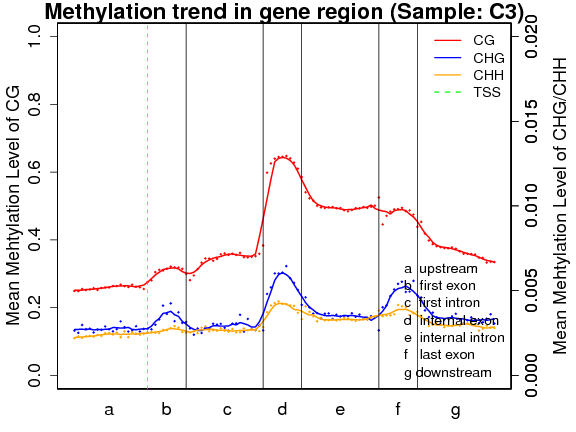

Supplement: Supplementary file 1 [file genes-11-01020-s001.zip › Supplemental Files/Supplemental Figures.docx]
